# Curcumin–Sodium Alginate and Curcumin–Chitosan Conjugates as Drug Delivery Systems: An Interesting Rheological Behaviour

**DOI:** 10.3390/molecules28155893

**Published:** 2023-08-05

**Authors:** Giuseppe Cirillo, Manuela Curcio, Cesare Oliviero Rossi, Giovanni De Filpo, Mariafrancesca Baratta, Michele De Luca, Francesca Iemma, Fiore Pasquale Nicoletta

**Affiliations:** 1 Department of Pharmacy, Health and Nutritional Sciences, University of Calabria, 87036 Rende (CS), Italy; manuela.curcio@unical.it (M.C.); michele.deluca@unical.it (M.D.L.); francesca.iemma@unical.it (F.I.); fiore.nicoletta@unical.it (F.P.N.); 2 Department of Chemistry and Chemical Technologies, University of Calabria, 87036 Rende (CS), Italy; cesare.oliviero@unical.it (C.O.R.); giovanni.defilpo@unical.it (G.D.F.); mariafrancesca.baratta@unical.it (M.B.)

**Keywords:** polyphenol conjugates, rheological measurements, sustained release, chitosan, sodium alginate

## Abstract

The conjugation of polyphenols is a valuable strategy with which to confer tailored properties to polymeric materials of biomedical interest. Within this investigation, we aim to explore the possibility to use this synthetic approach to increase the viscosity of conjugates, thus allowing the release of a loaded therapeutic to be better controlled over time than in neat polyphenols. Curcumin (CUR) was conjugated to sodium alginate (CA) and chitosan (CS) with functionalisation degrees of 9.2 (SA-CUR) and 15.4 (CS-CUR) mg g^−1^. Calorimetric analyses showed higher degrees of chain rigidity upon conjugation, with a shift of the degradation peaks to higher temperatures (from 239 to 245 °C and from 296 to 303 °C for SA-CUR and CS-CUR, respectively). Rheological analyses were used to prove the enhanced interconnection between the polymer chains in the conjugates, confirmed by the weak gel parameters, *A* and *z*. Moreover, the typical non-Newtonian behaviour of the high-molecular-weight polysaccharides was recorded, together with an enhancement of the activation energy, *Ea*, in CS-CUR vs. CS (opposite behaviour recorded for SA-CUR vs. SA). The evaluation of the delivery performance (of Doxorubicin as a model drug) showed sustained release profiles, opening opportunities for the development of controlled delivery systems.

## 1. Introduction

Over the last decades, several delivery technologies have been developed to address the pharmacokinetic concerns arising from the low water solubility, poor permeability, and unfavourable distribution/metabolism/excretion pathways of most active pharmaceutical ingredients [1,2]. Polymeric materials are widely recognised to possess suitable physico-chemical and biological features for facilitating the administration of biologically active molecules [3], allowing the attainment of an effective concentration at the proper site for a sufficient period of time to elicit the desired response [4,5]. Thus, in order to modulate the amount, site, and rate of drug release, there is a tremendous interest in the development of polymeric formulations and delivery devices with various architectures, including nanoparticle, micelle and vesicle systems, dendrimers, films, prodrugs, gels, and hydrogels [6,7,8].

Natural, synthetic, and semisynthetic polymers can improve the therapeutic outcomes of the dosage form prolonging the retention of the drug within the administration site through the modulation of the rheological properties, mainly in terms of increased viscosity and mechanical stability [9,10,11]. More recently, the incorporation of polymer conjugates with tailored biological activities (e.g., antioxidant and anti-inflammatory ones) within a pharmaceutical formulation was proven as a valuable strategy to allow the key features of the polymeric materials to be coupled with the biological peculiarities of the conjugate molecules [12,13,14]. Via this approach, some key issues of drug delivery can be addressed, including the low chemical and storage stability of some therapeutics of clinical interest [15,16], as well as the oxidative stress/inflammation induced by device–tissue interactions [17]. Natural polysaccharides show favourable characteristics for the preparation of drug delivery systems, including the presence of functional groups (i.e., amino, hydroxyl, and carboxyl groups) suitable for tailored chemical functionalisation, and the ability to interact with biological tissues via noncovalent bonding [18,19], although they possess some undeniable limitations due to their significant natural variability, complex synthetic routes, and poor mechanical properties [20,21].

We previously proved the suitability of polyphenol compounds as derivatising agents for the synthesis of polymer conjugates via an eco-friendly and biocompatible method involving the use of laccase as a biocatalyst [22], with the reaction consisting of the nucleophilic attack of nitrogen/oxygen-rich groups in the side chains of polymer materials on enzyme-oxidised polyphenols. As a result, we were able to prepare several bioconjugates with strong biological activities, suitable to be formulated into highly effective biomedical devices, such as anticancer and antimicrobial drug carriers [23], as well as being suitable as support for cell regeneration and protein crystallisation [24].

The rationale behind the present study lies in the chemical structure of a polyphenol species that, possessing multiple sites suitable for the reaction with laccase, allows a hypothesis of the crosslinking-like activity of the polyphenol. The polyphenol conjugate, indeed, can react with nucleophilic groups of two different polymer chains, leading to a stronger interconnection between them. Since a stronger interconnection may affect the rheological behaviour of the polyphenol bioconjugate, including its viscosity and activation energy, we extensively investigated such parameters by considering two polysaccharides widely used in biomedical fields, sodium alginate (SA) and chitosan (CS), functionalised with curcumin (CUR) using laccase chemistry.

This choice was based on the well-known and highly recognised applicability in the biomedical field of each species.

CUR is a yellow pigment extracted from *Curcuma longa*, an aromatic plant from the ginger family (*Zingiberaceae*), historically used as a spice and biological dye, but widely known for its interesting biological properties [25]. From a chemical point of view, CUR has two aromatic rings symmetrically substituted by methoxy and a phenolic OH groups in the *ortho* position, which are connected to a conjugated seven-membered hydrocarbon chain with an enone part and a 1,3-diketone group [26]. The biological activities of CUR, arising from its capability to bind various cell-signalling molecules [27], include antioxidant, anticancer, pro-apoptotic, antibacterial, antiparasitic, antimalarial, anti-inflammatory, wound-healing, neuroprotective, cardioprotective and radioprotective effects [28,29,30]. In cancer treatment, CUR is able to prevent cancer development and progression by inhibiting both the initiation of cancer and malignant cell proliferation during the promotion and progression of carcinogenesis [31,32].

SA, an edible heteropolysaccharide abundantly available in brown algae (*Phaeophyceae*), is a copolymer consisting of (1,4)-linked β-d-mannuronate and α-l-guluronate residues organised as consecutive G residues, consecutive M residues, and alternating M and G residues [33]. It is widely used in the biomedical field as a base material for the preparation of non-toxic hydrogel systems with outstanding properties such as a high-water content, soft consistency, high biocompatibility and biodegradability [34]. Such properties allowed SA-based biomaterials to be suitable candidates for application in tissue engineering, wound-healing, and drug delivery applications [35,36,37].

CS, a deacetylated form of chitin, is a linear homopolymer constituted by β-(1,4)-linked N-acetyl-glucosamine units, extensively found as a major component of crustacean and insect exoskeletons, and bacterial and fungal cell walls [38]. The superior biological properties of CS, including its biocompatibility, hemocompatibility, biodegradability, and muco-adhesion, make this polysaccharide a widely explored material in biomedicine, where it exerts antitumour and antioxidant activities, as well as wound healing efficacy, and bacteriostatic effects [39,40]. Moreover, the hydroxyl and amino groups in the CS side chains are active sites for various chemical reactions, including alkylation, acylation, and esterification, tailoring its physical and chemical properties, as well as biological activities [38].

Therefore, in order to enlarge the range of possible applications of such polysaccharides, we investigated some physico-chemical properties of SA-CUR and CS-CUR conjugates obtained via the reaction between the OH and NH_2_ groups present in SA and CS side chains with CUR. In particular, we were interested in how the different reactivities of the two polysaccharides with CUR could affect the rheological behaviour of the obtained conjugates and, consequently, their performance in releasing Doxorubicin (DOX), which was used as a model drug. Furthermore, the release profiles were analysed by applying suitable mathematical modelling.

## 2. Results and Discussion

### 2.1. Synthesis of Alginate and Chitosan Conjugates

Our research activity successfully proved that laccase (LCS) chemistry was a valuable tool for the conjugation of polyphenols, such as Catechin, Caffeic Acid and Curcumin, to both protein and polysaccharide materials, including Human Serum Albumin, Gelatin, Alginate, and Dextran [24,41,42,43]. In all cases, the enzyme was preliminary immobilised into acrylate hydrogel films for the better handing of the biocatalyst and to facilitate the purification steps [22]. It is well-known, indeed, that enzymes are valid and well-performing alternatives to common chemical catalysts, since they work under mild conditions with high specificity and without the need for toxic organic solvents. Nevertheless, their applicability in industrial processes is often hindered by the difficulties in recovery and recycling, as well as by the possibility to contaminate the final products with protein residues [44,45]. Enzyme immobilisation on solid supports (acrylate hydrogels in our case) was proven to be a valuable strategy to address these concerns, since purification and reuse could consist in the simple removal of the support [46,47]. It should be underlined that enzyme immobilisation, although allowing a bioconjugate with superior biological properties to be synthesised, carries out to an enhancement of the steric hinderance around polyphenol molecules, with a functionalisation degree being significantly affected by the limited mobility of the polymers in solution which, in turn, strongly depends on their molecular weights. This statement can be confirmed by comparing the functionalisation degree of bioconjugates (expressed in terms of mg of polyphenol per gram of conjugate) obtained by reacting Dextrans with different molecular weights (namely 6000 and 40,000 Da) with polyphenols via immobilised laccase chemistry; a 6000 Da sample was characterised by a functionalisation degree of 128 mg g^−1^, while a value of 23.9 mg g^−1^ was obtained when 40,000 Da Dextran was used [43,48]. Thus, since the aim of the present investigation is to enhance the functionalisation degree of the polymeric materials, we used the free form of the enzyme as a catalyst, which could remove, or at least reduce, the steric hinderance around the catalytic sites. Via this approach, a further reaction of the conjugate with the enzyme could be hypothesised, thus allowing an increase in the interconnection between two adjacent polymer chains. This is an extension of the supramolecular gel concept, where polyphenol compounds can be considered gelator agents promoting the formation of a 3D network via the self-assembly of fibrillar aggregates and subsequent strong interconnections among polymer chains via multiple non-covalent interactions, such as hydrogen bonding, van der Waals interactions, hydrophobic forces, and π-π stackings [49].

**Figure 1:**
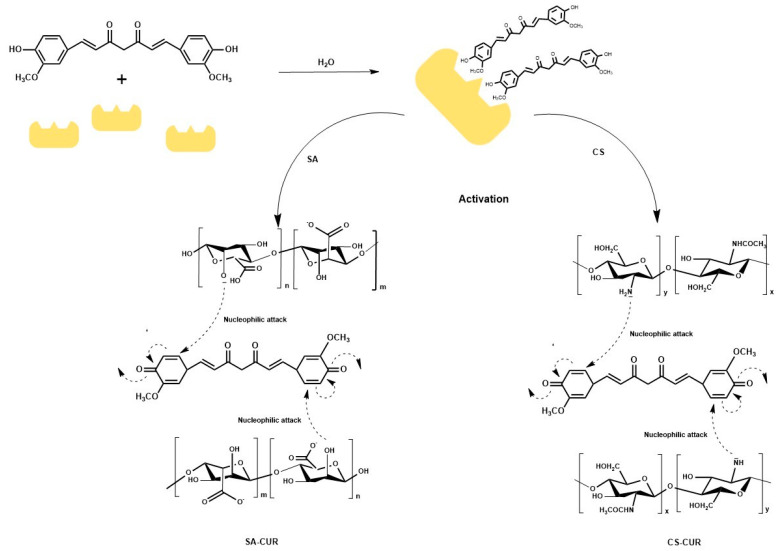
Schematic representation of the synthesis of SA-CUR and CS-CUR. Laccase activates CUR via oxidation and then the nucleophilic coupling of SA/CS allows the obtainment of the bioconjugates.

The synthesis of the SA and CS conjugates, labelled SA-CUR and CS-CUR, respectively, is illustrated in Figure 1.

As reported in the literature, the conjugation reaction is composed of two steps, with the enzyme being involved in the first one. In detail, laccase catalyses the one-electron oxidation of the polyphenol with the formation of a reactive *o*-quinones [50], undergoing a complex set of non-enzymatic reactions with the nucleophilic groups in the polysaccharide side chains [51]. The exact chemistry behind this set of reactions is not well-understood, but can be described as a Michael-type reaction resulting in the covalent coupling of CUR residues to polysaccharide chains [52].

**Figure 2:**
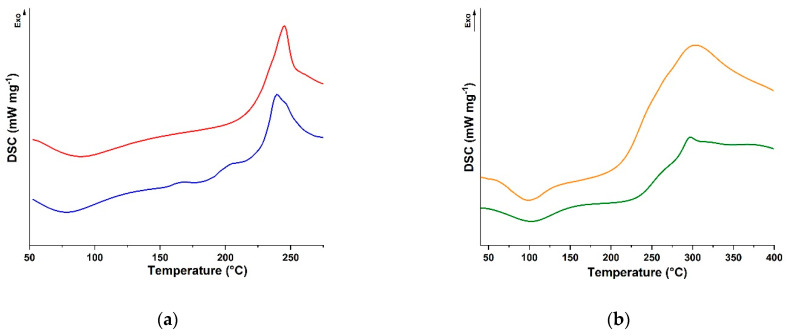
DSC curves of (**a**) SA (blue) and SA-CUR (red), and (**b**) CS (green) and CS-CUR (orange).

The synthetic procedure was optimised by maximising the amount (by weight) of reactants to be dispersed in the reaction feed, considering that the reaction media contained a trace (2.0%) of DMSO to promote the solubilisation of CUR without affecting that of both polysaccharides, as well as that of acetic acid (1.0%) when CS was used as the polymeric counterpart. Some key considerations should be made for the characterisation of the conjugates. Although ^1^H-NMR is one of the leading techniques for providing information about the structural properties of small organic and inorganic molecules, hybrid compounds, macromolecules, and biological samples, it is not feasible in this context. The intrinsic viscoelastic properties of either physically or chemically cross-linked networks, indeed, dramatically restricts tumbling, thereby reducing molecular motion, and affects chemical shift anisotropy and dipolar and quadrupolar interactions. This results in the appearance of broad signals in the solution-state NMR spectra and makes challenging the extraction of clear information related to chemical and structural details [53]. Thus, to assess the derivatisation degree of SA-CUR and CS-CUR conjugates, we used an indirect methodology based on the determination of the polyphenol moieties (mg polyphenol per gram of conjugate) via the Folin–Ciocalteu assay [24], obtaining functionalisation degrees of 9.2 and 15.4 mg g^−1^ for SA-CUR and CS-CUR, respectively. This difference could be attributed to the different nucleophilic character of the reactive groups in the polysaccharide side chains, and to the presence of nitrogen-rich groups in CS in particular. Moreover, to confirm our hypothesis that the use of free laccase could increase the functionalisation degree, we tested the use of immobilised laccase as a catalyst for the conjugation reactions, reaching, as expected, lower values (of 2.1 and 8.6 mg g^−1^ for SA-CUR and CS-CUR, respectively). Further characterisation of the conjugates was performed in terms of the determination of the calorimetric properties via DSC analysis (Figure 2).

It was found that the presence of the CUR moieties led to a shift of the exothermal degradation peak to higher temperatures (from 239 to 245 °C and from 296 to 303 °C for SA and CS, respectively), which is consistent with a higher degree of chain rigidity upon conjugation [54].

### 2.2. Rheological Characterisation of Conjugates

Before testing the suitability of the SA-CUR and CS-CUR conjugates as delivery systems able to modulate the time of release of a loaded therapeutic by virtue of the modified chemical structure of the two polysaccharides upon conjugation with CUR, we determined the rheological behaviour of both SA-CUR and CS-CUR. The core idea of the manuscript is to check that an increase in the interconnections between the polysaccharide chains induced by CUR conjugation can affect the viscosity of the conjugates and thus their delivery performance. Several literature data, indeed, indicated that the rheological properties of vehicles influence the drug release profiles of drugs, with more viscous behaviour often resulting in more sustained release [55]. On the other hand, a suitable balance between viscosity and handling should be reached, tailoring the composition of the dosage form to specific administration routes (e.g., ophthalmic, oral, intra-articular, vaginal, etc.) [56,57].

**Figure 3:**
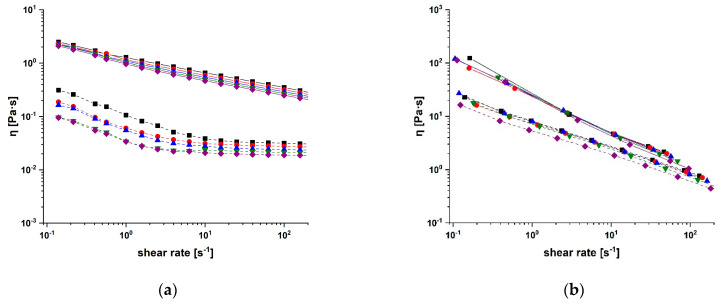
Viscosity versus shear rate for (**a**) SA (dashed lines) and SA-CUR (solid lines), and (**b**) CS (dashed lines) and CS-CUR (solid lines) at 25 (■), 30 (●), 35 (▲), 40 (▼), and 45 (♦) °C.

In our experimental protocol, we firstly assessed the variation in viscosity, *η*, versus that in shear rates, for samples at different temperatures (Figure 3).

In all cases, the typical non-Newtonian behaviour (pseudoplastic/shear thinning) of the high molecular weight polyelectrolyte biopolymers was observed [58], with the viscosity decreasing at higher shear rates [59]. This agrees with literature data reporting a higher ordering of the polymer chains upon an increase in shear rate values, leading to more oriented chains and thus to lower viscosity values [60]. It is also evident that conjugation with CUR increased the viscosity values for both SA-CUR and CS-CUR of one logarithm unit (at lower shear rates), as a result of increased interactions between the anisometric polymer chains [61].

**Figure 4:**
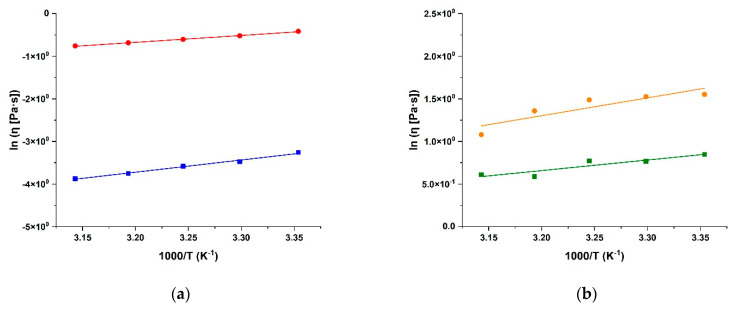
Arrhenius plots of the suspension viscosity for (**a**) SA (blue) and SA-CUR (red), and (**b**) CS (green) and CS-CUR (orange) for shear rate (10^1^ s^−1^). Linear fits were used for connecting the data.

Moreover, the temperature was found to affect the relative viscosity of native polysaccharides, in a greater way than it did for bioconjugates. In general, viscosity decreases with an increasing temperature, with an Arrhenius-like behaviour (Equation (1)) [58].

(1) $$\eta =\eta_{0}e^{{(E}_{a}/RT)}$$

where *η*_0_, the zero-shear viscosity parameter, represents the viscosity at an “infinite temperature”, *E_a_* is the activation energy of the viscous flow, defined as the energy required for the fluid molecules to move freely [60], and *R* is the universal gas constant. The fitting of the experimental data recorded at a shear rate of 10^1^ s^−1^ is depicted in Figure 4.

The comparison of the *E_a_* values of SA and CS with those recorded for the corresponding bioconjugates proved the interesting and opposite behaviour for the two conjugates (Table 1).

The modification of polymer chains by CUR moieties, indeed, resulted in an enhancement of the *E_a_* of CS-CUR vs. CS, but resulted in a decrease of this value in SA-CUR vs. SA. The CS-CUR behaviour can be easily explained via the hypothesis of the existence of stronger restrictions in the freedom of the biopolymeric chains’ movement in CS-CUR rather than that in CS, due to stronger intermolecular entanglement upon CUR conjugation [62]. On the other hand, the decreased *E_a_* values recorded in the SA-CUR conjugate vs. pristine SA can be explained by the formation of “cooperatively rearranging regions”, which can be considered clusters of particles tending to move collectively easily with an increasing temperature [63].

**Figure 5:**
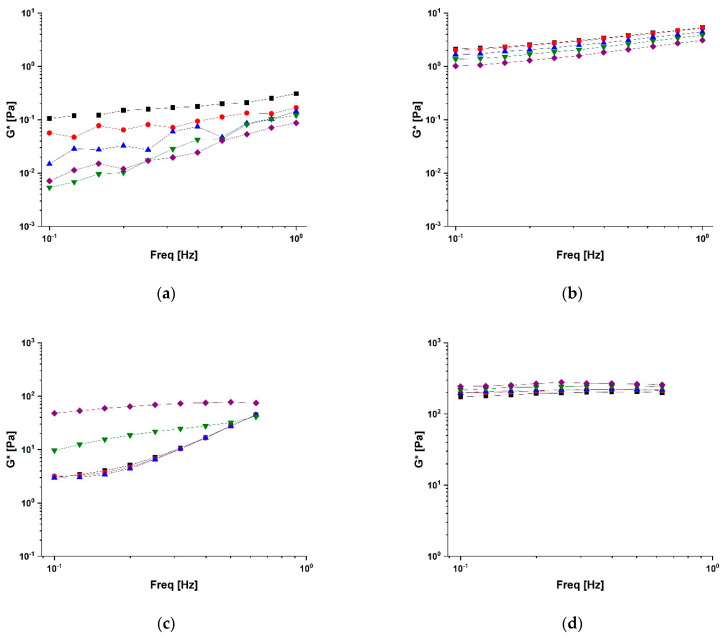
Complex shear modulus (G*) for (**a**) SA, (**b**) SA-CUR, (**c**) CS, and (**d**) CS-CUR at 25 (■), 30 (●), 35 (▲), 40 (▼), and 45 (♦) °C.

All of these data suggested that the conjugation increased the interconnection between the polymer chains, with the formation of a stronger network in the CS-CUR case, while agglomerates or flocs of the bioconjugate were obtained when SA was used as base polysaccharide [64], probably as a consequence of the lower nucleophilic behaviour of oxygen-rich SA with respect to the nitrogen-rich CS. The result of the analysis of the *η*_0_ values (Table 1) is consistent with this hypothesis, since higher *η*_0_ values imply higher chain interactions within polymeric materials [65]. To further confirm this statement, we performed small-amplitude oscillatory experiments at different temperatures and interpreted the data in accordance with the “weak-gel model” theory proposed by Bohlin and Winter, which gives a direct link between the microstructure of the material and its rheological properties [66]. In this model, the following equation, Equation (2), can be applied:

(2) $$G^{*}\left(\omega \right)=\sqrt{G^{\prime }{\left(\omega \right)}^{2}+G^{\prime \prime }{\left(\omega \right)}^{2}}=A\omega^{1/z}$$

where *G*′(*ω*) (the “storage” or “elastic” modulus) and *G*″(*ω*) (the “loss” or “plastic” modulus) are the real and imaginary components of the complex shear modulus, G*(*ω*). According to the model of Bohlin and Winter, *A* is a constant, which can be interpreted as the “interaction strength” between the flow rheological units, and *z* is the number of flow units interacting with each other to give the observed flow response. The plots of G* as a function of frequency at different temperatures are depicted in Figure 5.

The results of the analysis of the weak gel parameters collected in Table 2 clearly proved that the conjugation procedure led to a remarkable increase in both *A* and *z* values, thus suggesting an enhancement of the strength and number of the cohesive interactions among the polymer chains in bioconjugates vs. those in native polysaccharides with the increase in *z* values being remarkably higher for CS-CUR.

It is worth underlining the effect of temperature on this system: the higher the temperature, the larger the *A* and *z* values (from 217 to 263 and from 12 to 20, respectively), probably as a consequence of a gel-like formation induced by the temperature. This phenomenon was observed only for the CS-CUR system, while the SA-CUR values kept the *z* values quite constant in temperature, while *A* decreased as temperature increased This indicates that no structural change was induced by the temperature on SA-CUR, with only kinetic effects being shown.

Moreover, as expected, all weak gel data agree with the results of the rheological steady analysis where gel formation was observed too.

### 2.3. Drug Delivery Performance

The ability of a delivery device to control and modulate the release of loaded bioactive agents depends on multiple physico-chemical parameters, including, but not limited to, their composition, size and shape, surface properties, hydrophobic to hydrophilic balance, and rheological and mechanical properties [67,68]. With the aim to elucidate how the releasing behaviour can be affected by an increase in the viscosity of the polysaccharide materials due to the stronger interconnection between the polymer chains upon conjugation with CUR, we used Doxorubicin (DOX) as a model drug and recorded the release profiles at pH 5.5 as a function of time according to the following equation, Equation (3):

(3) $$DOX\;release=\frac{M_{t}}{M_{0}}$$

where *M_t_* and *M*_0_ are the drug amounts detected at time t and 0, respectively. The choice of pH 5.5 was due to the well-known pH responsivity of DOX release, which is widely accepted to be promoted in the acidic environment of cancer tissues [69]. On the other hand, an incubation time of 36 h was tested due to the nature of the polysaccharide materials used, which can undergo biodegradation in a physiological environment [70,71,72]. Moreover, the same drug to carrier ratio was explored to clearly correlate the releasing profiles to the different rheological behaviours of the conjugates, since the loading of different amounts can influence the release rates which cannot be directly related to the chemical nature of the proposed nanoformulations.

**Figure 6:**
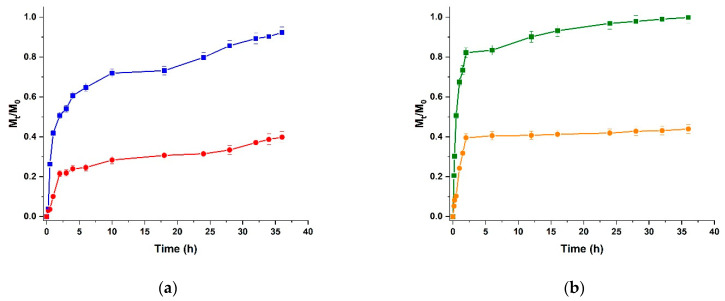
DOX release profiles at pH 5.5 from (**a**) SA (blue) and SA-CUR (red), and (**b**) CS (green) and CS-CUR (orange).

The release profiles obtained from conjugated polysaccharides were compared with those obtained using native polymers (Figure 6).

It is clearly evident that the increased interconnection between the polysaccharide chains due to CUR conjugation allowed the release profiles to be more controlled and prolonged overtime, with the maximum DOX release being reduced by 56% for both conjugates, suggesting a similar effect of the derivatisation reaction. More interestingly, the release from SA-CUR reached 40% after 36 h, while a faster release was obtained when CS-CUR was used as a hydrogel matrix (40% of release after 3 h). This difference could be attributed to the stronger affinity of DOX to SA at the pH value tested for the release. In these conditions, indeed, both species are in a protonated form and drug release can be hindered by the formation of new hydrogen bonds [73].

For a more extensive characterisation of the release behaviour, the experimental data were analysed by applying suitable mathematical models available in the literature, including the zero-order (Equation (4)), first-order (Equation (5)), and Peppas–Sahlin (Equation (6)) models, which were valid for *M_t_/M*_0_ < 0.95 [74]:

(4) $$\mathrm{Zero}-\mathrm{order}\;\;\;\;\frac{M_{t}}{M_{0}}=k_{0}t$$

(5) $$\mathrm{First}-\mathrm{order}\;\;\;\;\frac{M_{t}}{M_{0}}=a\left(1-e^{-k_{1}t}\right)$$

(6) $$\mathrm{Peppas}–\mathrm{Sahlin}\;\;\;\;\frac{M_{t}}{M_{0}}=k_{S}^{\prime }t^{m}+k_{S}^{\prime \prime }t^{2m}$$

Here, *t* is the release time; $k_{0},k_{1},k_{S}^{\prime }$, and $k_{S}^{\prime \prime }$ are the zero-order, first-order, Fickian diffusion, and anomalous diffusion kinetic constants; *a* is the first-order release coefficient, and *m* is the Peppas–Sahlin coefficient. The kinetic parameters obtained by applying such semi-empirical models are presented in Table 3.

The results proved that the release of DOX was not a steady-state phenomenon (R^2^ < 0.83 for Equation (4)) [75], but dependent on either the concentration gradient between the hydrogel and releasing media phases [76] or the drug diffusion from the polymer chains [77] (R^2^ > 0.90 for both Equations (5) and (6)). A more extensive analysis of the kinetic constants clearly showed the effect of CUR conjugation on drug release, further confirming the hypothesis that a stronger interconnection between the polymer chains resulted in more prolonged drug release. In detail, *k*_1_ values were reduced from 1.32 (CS) to 0.87 (CS-CUR) and from 0.42 (SA) to 0.32 (SA-CUR). The more relevant reduction recorded for CS vs. SA conjugate (34 vs. 24%) could be ascribed to the different behaviours of the parent polysaccharides, since the lower affinity of CS for the drug molecules led to a significantly faster release. On the other hand, the application of the Peppas–Sahlin model gave more information about the release mechanisms, which mainly include the Fickian diffusion of the drug molecules between the hydrogel chains. This was more evident in CS than in SA cases, since the higher DOX to SA affinity resulted better fitting (with higher R^2^ values recorded for both SA and SA-CUR with respect to CS and CS-CUR). Similarly, the lower $k_{S}^{\prime }$ values recorded for SA/SA-CUR indicate the more pronounced involvement of diffusion phenomena when SA was used as a base polysaccharide. In both cases, the higher $k_{S}^{\prime }\;vs.\;k_{S}^{\prime \prime }$ values allowed the hypothesis that the adopted synthetic strategy significantly affected the release kinetics, although it did not modify the main phenomena evoked to explain the release mechanism, which is that of Fickian diffusion as expected for any polymer gel system.

Thus, it can be stated that the use of CUR as a crosslinking-like agent to improve the interconnection between polysaccharide chains is a valuable strategy to enhance the viscosity of a polymeric solution which, in turn, can modulate the release of a loaded therapeutic. The conjugation enhanced the interconnections between the polymer chains, with the obtainment of a polymer network and an agglomerated polymer for CS and SA, respectively. In both cases, the conformation of conjugates hindered the diffusion of drug molecules from the hydrogel to the releasing media phases, thus allowing the obtainment of release profiles prolonged overtime, fulfilling the needs of sustained chronic therapy avoiding multiple administrations.

## 3. Materials and Methods

### 3.1. Laccase Immobilisation

The immobilisation of laccase (*Trametes versicolor*, EC 1.10.3.2) was carried out in accordance with our previous work [22]. Briefly, 534 mg (7.51 mmol) of Acrylamide and 466 mg (0.62 mmol) of Polyethylene Glycol Dimethacrylate750 were added to a laccase solution (25 mg in 3.0 mL sodium citrate buffer, 10^−3^ mol L^−1^, pH 5.0). Then, 2.4 (*w*/*w*) % 1-[4-(2-hydroxyethoxy)-phenyl]-2-hydroxy-2-methyl-1-propane-1-one (Irgacure 2959, maximum absorption at around 275 nm) was added as a photo-initiator and the mixture was placed in a reaction cell consisting of two 10 × 10 cm^2^ glass plates brought together using binder clips and separated with Teflon spacers (thickness 1.6 mm). The solution was allowed to react for 10 min under a high-pressure mercury lamp (HPK 125, Philips, Amsterdam, Netherlands, 10 mW cm^−2^, 275 nm wavelength 275). After removing unreacted species via washing with distilled water, the resulting hydrogel was dried overnight in an oven under vacuum at 40 °C.

Irgacure 2959 was purchased from BASF, Ludwigshafen, Germany, and all other chemicals were from Merck KGaA, Darmstadt, Germany.

### 3.2. Synthesis of Bioconjugates

SA-CUR and CS-CUR conjugates were synthesised in accordance with the literature with some modifications [48]; 250 mg of polysaccharides and 10 mg of CUR were dissolved in 10 mL H_2_O (containing 1% CH_3_COOH in the case of CS)/DMSO mixture 90/10 by vol and reacted by adding free laccase (0.25 U) at 37 °C under 70 rpm for 12 h. After the reaction time, the conjugates were purified via a dialysis process (dialysis tubes of 6–27/32″ Medicell International LTD, MWCO: 12,000–14,000 Da) and dipped into a glass vessel containing distilled water at room temperature for 72 h, until the complete removal of unreacted CUR. The bioconjugates were collected after a freeze-drying process was carried out using a freeze drier (Micro Modulyo, Edwards Lifesciences Corporation, Irvine, CA, USA). The amount of CUR in the washing media was determined using high-pressure liquid chromatography (HPLC) equipment including a Jasco PU-2089 Plus liquid chromatograph equipped with a Rheodyne 7725i injector (fitted with a 20 μL loop), a Jasco UV-2075 HPLC detector operating at 420 nm, a Jasco-Borwin integrator (Jasco Europe s.r.l., Milan, Italy) and a Tracer Excel 120 ODS-A column with a particle size of 5 μm, 15 × 0.4 cm (Teknokroma, Barcelona, Spain); the mobile phase was methanol at a flow rate of 1.0 mL min^−1^.

The same procedure was adopted when immobilised laccase was used as a biocatalyst.

### 3.3. Characterisation Procedure

Calorimetric analyses were carried out using a DSC200 PC differential scanning calorimeter (Netzsch, Selb, Germany). Using a standard procedure, about 5.0 mg of the dried sample was placed in an aluminium pan, and then sealed tightly using an aluminium lid. The thermal analyses were performed from 50 to 300 °C (SA and SA-CUR) and from 50 to 400 °C (CS and CS-CUR) under a dry nitrogen atmosphere with a flow rate of 25 mL min^−1^ and a heating rate of 5 °C min^−1^.

The rheological analyses were conducted using a shear strain-controlled rheometer, RFS III (TA Instruments, New Castle, DE, USA), equipped with a parallel plate (*ϕ* = 25 mm) and a concentric cylinder (inner radius: 17 mm; gap: 1.06 mm). The temperature was controlled using a Peltier apparatus (±0.1 °C). To prevent errors due to evaporation, the measuring geometries were surrounded by a solvent trap containing water. The solutions submitted to shearing rates during experiments were fully transparent and free from foam and air bubbles. Two set of experiments were performed: (i) steady flow experiments in a shear rate range of 10^−1^–2.5 10^2^ s^−1^; (ii) dynamic shear experiments in a frequency range of 10^−1^–1.0 Hz.

The amount of available phenolic groups was calculated in accordance with the Folin–Ciocalteu procedure [24] and expressed in mg of CUR per gram of polymer using the equation obtained from the calibration curve of the free CUR. Briefly, in separate experiments, 25 mg of the SA-CUR and CS-CUR conjugate was dissolved in 6.0 mL of distilled water and then 1.0 mL of the Folin–Ciocalteu reagent was added. After 3 min of stirring, 3 mL of Na_2_CO_3_ (7.5%) was added and the mixture was allowed to stand for 2 h with intermittent shaking. The absorbance was measured at 760 nm against a blank polysaccharide solution prepared in the same condition on an Evolution 201 spectrophotometer (ThermoFisher Scientific, Hillsboro, OR, USA).

### 3.4. In Vistro Drug Release

In separate experiments, 0.25 mg of DOX was added to 25 mg of SA, SA-CUR, CS, and CS-CUR in 2.5 mL of distilled water for 24 h. The release experiments were performed in phosphate-buffered saline at pH 5.5 in a dialysis bag (MWCO: 12,000–14,000 Da), and it was dialyzed against fresh PBS (12.5 mL). At predetermined time intervals, the amount of DOX in the releasing media was determined via UV-Vis on a Jasco V-530 UV/Vis spectrometer (Jasco Europe s.r.l., Milan, Italy) at 496 nm. From the calibration curves of DOX in PBS (pH 7.4 and pH 5.0), the cumulative amount of drug released was calculated using the following equation, Equation (7):

(7) $$DOX\;release=\frac{M_{t}}{M_{0}}$$

where *M_t_* and *M*_0_ are the amounts of the drug in the solution at time t and loaded into the carrier, respectively. Experiments were performed in triplicate and the results are expressed as means ± SD.

All chemicals were from Merck KGaA (Darmstadt, Germany).

### 3.5. Statistical Analyses

Experiments were performed in triplicate and data expressed as mean ± S.E.M.

## 4. Conclusions

We provided experimental evidence that the conjugation of polyphenol moieties (i.e., Curcumin) to polysaccharide materials (i.e., sodium alginate and chitosan) via laccase chemistry is a valuable approach to modify the rheological behaviour of the obtained conjugates (i.e., enhancement of the viscosity of the resulting polymer solutions), thus allowing the modulation of the releasing profiles of a loaded of therapeutic agent (i.e., Doxorubicin). It was hypothesised that the nucleophilic coupling of oxygen- and/or nitrogen-rich groups in polysaccharide side chains allowed the interconnection between the polymer chains to be increased, with CUR molecules acting as a bridge between two adjacent polymer chains. Interestingly, the conjugation reaction led to different behaviours due to the different reactivities of SA (which is oxygen-rich) and CS (which is nitrogen-rich); a stronger network was obtained when CS was used as base material, while the involvement of SA allowed the formation of agglomerates or flocs of the bioconjugate. In both cases, conjugation was responsible for the enhanced viscosity, as assessed via extensive rheological investigations, leading to the DOX releasing profile being extended overtime, with potential key advantages in clinical practice. Although further investigations are required for more extensive characterisation and application in suitable ex vivo and in vivo models, taking together the results here presented makes these systems promising carriers for application in the biomedical field.

## Figures and Tables

**Table 1:** *η*_0_, *Ea*, and R^2^ values obtained using the Arrhenius equation from shear measurements for native and conjugated polysaccharides.

Sample	R^2^	*η*_0_ (Pa s)	*E_a_* (kJ mol^−1^)
SA	0.9903	(2.5 ± 0.1) 10^−6^	23.8 ± 0.1
SA-CUR	0.9968	(2.9 ± 0.1) 10^−3^	10.3 ± 0.1
CS	0.9460	(2.6 ± 0.1) 10^−2^	13.4 ± 0.1
CS-CUR	0.9081	(4.4 ± 0.1) 10^−2^	17.4 ± 0.1

**Table 2:** Weak gel parameters for native and conjugated polysaccharides. Data are expressed as mean ± S.E.M.

Sample	Temperature	R^2^	*A* (±0.1) $\left(Pa\;s^{1/z}\right)$	*z* (±0.1)
SA	25	0.9831	0.3	1.6
	30	0.9819	0.2	1.4
	35	0.9471	0.1	1.2
	40	0.9754	0.1	1.2
	45	0.9527	0.1	1.2
SA-CUR	25	0.9946	5.6	2.1
	30	0.9943	4.7	2.1
	35	0.9958	4.0	2.0
	40	0.9748	3.0	2.3
	45	0.9964	2.7	2.3
CS	25	0.9972	127.2	0.6
	30	0.9946	112.2	0.6
	35	0.9983	115.5	0.5
	40	0.9937	102.1	0.5
	45	0.9550	116.9	0.6
CS-CUR	25	0.8688	217.2	11.9
	30	0.8482	229.7	16.9
	35	0.8469	231.5	18.0
	40	0.8461	246.0	20.4
	45	0.8093	263.4	19.6

**Table 3:** Kinetic parameters for DOX release from native and conjugated polysaccharides. Data fall within 5% S.E.M.

Sample	Zero Order		First Order		Peppas-Sahlin			
	R^2^	*k*_0_ (s^−1^)	R^2^	*k*_1_ (s^−1^)	R^2^	*m*	$k_{S}^{\prime }$ (s^−m^)	$k_{S}^{\prime \prime }$ (10^−1^ s^−2m^)
SA	0.7950	0.03	0.9273	0.42	0.9569	0.40	0.40	0.45
SA-CUR	0.8288	0.01	0.9379	0.32	0.9446	0.45	0.13	0.12
CS	0.6813	0.04	0.9756	1.32	0.9455	0.33	0.71	1.31
CS-CUR	0.6950	0.02	0.9850	0.87	0.9083	0.42	0.27	0.39

## Data Availability

Data are available from the authors.

## Sample Availability

Samples are available from the authors.
